# Tolerability of the Oscar 2 ambulatory blood pressure monitor among research participants: a cross-sectional repeated measures study

**DOI:** 10.1186/1471-2288-11-59

**Published:** 2011-04-27

**Authors:** Anthony J Viera, Kara Lingley, Alan L Hinderliter

**Affiliations:** 1 Department of Family Medicine, University of North Carolina at Chapel Hill, Chapel Hill, North Carolina, USA; 2 Department of Medicine, Division of Cardiology, University of North Carolina at Chapel Hill, Chapel Hill, North Carolina, USA

## Abstract

**Background:**

Ambulatory blood pressure monitoring (ABPM) is increasingly used to measure blood pressure (BP) in research studies. We examined ease of use, comfort, degree of disturbance, reported adverse effects, factors associated with poor tolerability, and association of poor tolerability with data acquisition of 24-hour ABPM using the Oscar 2 monitor in the research setting.

**Methods:**

Sixty adults participating in a research study of people with a history of borderline clinic BP reported on their experience with ABPM on two occasions one week apart. Poor tolerability was operationalized as an overall score at or above the 75th percentile using responses to questions adapted from a previously developed questionnaire. In addition to descriptive statistics (means for responses to Likert-scaled "0 to 10" questions and proportions for Yes/No questions), we examined reproducibility of poor tolerability as well as associations with poor tolerability and whether poor tolerability was associated with removal of the monitor or inadequate number of BP measurements.

**Results:**

The mean ambulatory BP of participants by an initial ABPM session was 148/87 mm Hg. After wearing the monitor the first time, the degree to which the monitor was felt to be cumbersome ranged from a mean of 3.0 to 3.8, depending on whether at work, home, driving, or other times. The most bother was interference with normal sleeping pattern (mean 4.2). Wearers found the monitor straightforward to use (mean 7.5). Nearly 67% reported that the monitor woke them after falling asleep, and 8.6% removed it at some point during the night. Reported adverse effects included pain (32%), skin irritation (37%), and bruising (7%). Those categorized as having poor tolerability (kappa = 0.5 between sessions, p = 0.0003) were more likely to report being in fair/poor health (75% vs 22%, p = 0.01) and have elevated 24-hour BP average (systolic: 28% vs 17%, p = 0.56; diastolic: 30% vs 17%, p = 0.37). They were also more likely to remove the monitor and have inadequate numbers of measurements.

**Conclusions:**

The Oscar 2 ABPM device is straightforward to use but can interfere with sleep. Commonly reported adverse effects include pain, skin irritation, and bruising. Those who tolerate the monitor poorly are more likely to report being in fair or poor health and to remove it, particularly at night.

## Background

The evidence for the clinical utility of ambulatory blood pressure monitoring (ABPM) continues to accumulate. Blood pressure (BP) measured by this technique is more closely associated with prognosis and is considered to be the current gold standard method for determining an individual's true BP [1,2]. Clinically, ambulatory BP monitoring is valuable for confirming suspected white-coat hypertension or evaluating white-coat effect in patients with apparently difficult-to-control hypertension, for detecting masked hypertension, and for gaining a better estimate of BP control among treated patients [2-4]. Additionally, ABPM is useful when evaluating symptoms possibly due to hypotension and is the only technique currently available to assess night-time (sleep) BP [2]. In addition to its clinical usefulness, ambulatory BP monitoring is now recognized as critical in studies of BP-lowering treatments [5].

An obvious downside of ambulatory BP monitoring is the potential inconvenience, related to the need to keep the cuff on the arm during the entire measurement period (usually 24 hours) as well as having to wear the monitor unit on the waist (by a belt or strap) during the day and keep it at the bedside at night. It is thus important to have some sense of the individual's experience when undergoing ambulatory BP monitoring. The Oscar 2 (SunTech Medical, Morrisville, NC) ambulatory BP monitor, in addition to being light and compact, uses motion tolerant algorithms to reduce re-inflates and failed readings. It has an additional feature (auto-intelligent inflation pressure) designed to reduce measurement time and reduce patient discomfort by controlling cuff inflation. A special cuff (the Orbit cuff) has a stretch-sleeve designed to maintain cuff placement and promote patient comfort. We assessed tolerability of wearing the Oscar 2 ambulatory BP monitor among a group of adults not yet diagnosed with hypertension who participated in a research project. A unique feature of our study is that participants wore the monitors on two occasions and they reported on their experiences after each monitoring session.

## Methods

This study was part of a project conducted to assess the reproducibility of classifications of blood pressure based on pairings of office and out-of-office measurements for purposes of informing the design of a larger study. We recruited sixty adults via signs inviting participation in a study of people with a recent clinic BP measurement that was "borderline" or "a little high." Signs were posted in an academic family medicine center, two community family medicine practices and in a clinical research center. Individuals interested in participating contacted a study coordinator to confirm eligibility and schedule their study visits. To be eligible, a person had to be 30 years of age or older (although one person who was actually 29 years old ended up being enrolled), have no diagnosis of hypertension and be on no medications to lower BP. Exclusion criteria included pregnancy, dementia, any condition that would preclude wearing the monitor (including an arm circumference >46 cm), and persistent atrial fibrillation or other arrhythmia. As an incentive, participants were offered $150 for completion of the study.

Informed consent was obtained prior to any study procedures. Participants underwent two 24-hour ambulatory BP monitoring sessions one week apart using the Oscar 2 oscillometric monitor. The Oscar 2 has been validated for use in adults by both the British Hypertension Society protocol and the International Protocol for the validation of blood pressure measuring devices [6,7]. The monitors were programmed to measure BP at 30 minute intervals during the daytime (6 am to 11 pm) and at 1 hour intervals during the nighttime (11 pm to 6 am). Maximum BP measurement time was limited to less than 140 seconds, and the monitors were set for a maximum pressure of 220 mm Hg. Participants were given verbal instructions on wearing the monitor, including that that they should try to leave the cuff on during the entire monitoring period, that they should try to hold their cuffed arm as still as possible during a reading to ensure that the monitor would get an accurate reading, that cuff inflation would cause a tight feeling around the arm, and that faulty readings would trigger a repeat measurement. Following each monitoring session, participants completed a questionnaire to assess tolerability of wearing the monitor (Appendix). This questionnaire was adapted from one used in a previous study designed to assess patient satisfaction with wearing an ambulatory BP monitor [8].

We report responses to Likert-scaled questions as means with standard deviations and responses to categorical questions as percentages. We created an overall tolerability score as the sum of the responses to questions 1 and 4 through 8 minus the responses to questions 2 and 3. We defined poor tolerability as a total score of Likert-scaled responses at or above the 75th percentile of the sample. We examined agreement on poor tolerability between the first and second sessions of wearing the monitor and calculated the kappa coefficient and its p-value. We then examined bivariate associations of demographic and general health characteristics with poor tolerability at first wearing of the monitor and tested for significance using chi-square. Finally, we examined whether poor tolerability was associated with reported removal of the monitor and inadequate number of BP measurements. Inadequate number of measurements was defined as <14 usable readings for daytime and <7 usable readings fro nighttime. This study was approved by the Office of Human Research Ethics at the University of North Carolina.

## Results

### Participant characteristics

The mean (±SD) age of the sixty participants was 47.6 (±10.5). Most participants were between 45 and 64 years (56.7%) or between 29 and 44 years (36.7%) (Table 1). A small proportion was older than 65 years (6.7%). Slightly more than half were female. Approximately 57% were white, and 40% were Black. Few were of Hispanic ethnicity. Over half were college graduates, and the majority (91.7%) reported good to excellent health. Most (83.3%) were also nonsmokers and overweight (25.0%) or obese (56.7%). Approximately two-thirds were married or living with a partner. Everyone who enrolled in the study completed it, although not everyone provided complete data. The sample mean of the average 24-hour ambulatory BPs of participants was 148/87 mm Hg from the first sessions and 146/85 mm Hg from the second sessions.

**Table 1:** Participant characteristics (N = 60)

Characteristic	n	Percent*	Mean (SD)
Age group (years)			
29-44	22	36.7	
45-64	34	56.7	
>65	4	6.7	
Female sex	31	51.7	
Race			
Black	24	40.0	
White	34	56.7	
Other	2	3.3	
Hispanic ethnicity	2	3.3	
Education level			
Some high school	2	3.3	
High school graduate	3	5.0	
Some college	21	35.0	
College graduate	34	56.7	
Self-reported health			
Excellent	10	16.7	
Very good	29	48.3	
Good	16	26.7	
Fair or poor	5	8.3	
Nonsmoker	50	83.3	
BMI			
Normal (<25 kg/m^2^)	11	18.3	
Overweight (25-29 kg/m^2^)	15	25.0	
Obese (≥30 kg/m^2^)	34	56.7	
Arm circumference**			
<35 cm	36	72.0	
≥35 cm	14	28.0	
Married or living with partner**			
Yes	35	65.0	
No	15	35.0	
24-hour systolic BP, mm Hg (1^st ^time)			148 (14)
24-hour diastolic BP, mm Hg (1^st ^time)			87 (10)
24-hour systolic BP, mm Hg (2^nd ^time)			146 (14)
24-hour diastolic BP, mm Hg (2^nd ^time)			85 (10)

BMI, body mass index

*Some percentages do not add exactly to 100 due to rounding

** missing data on 10 participants

### Monitor comfort, ease of use, and bother

After wearing the monitor the first time, the extent to which people found the monitor heavy was minimal, as indicated by a mean score of 1.4 on the 0 to 10 scale (Table 2). Wearers generally found the monitor straightforward to use (mean 7.5). However, the comfort of the monitor was not rated as favorably, with a mean score of 3.9. The degree to which the monitor was cumbersome to wear ranged from a mean of 3.0 to 3.8, depending on the location. The noise of the pump was not much of a bother to the wearer (mean ranged from 1.3 to 2.3 depending on location) or to others (mean 2.3). The most bother was due to interference with the normal sleeping pattern (mean 4.2). There was little change in mean responses from first to second wearing of the monitor. The only exceptions were that the extent to which the monitor was straightforward to use increased to 8.4 (p = 0.06), and it was rated slightly more embarrassing (mean 1.7 to 2.2, p = 0.04) and slightly more cumbersome to wear at work (mean 3.1 to 3.8, p = 0.06) during the second monitoring session.

**Table 2:** Monitor comfort, ease of use, and degree of bother

	**Mean (SD) after 1**^**st **^**session**	**Mean (SD) after 2**^**nd **^**session**	p-value*
Found monitor heavy	1.4 (2.1)	1.4 (1.9)	0.94
Found monitor comfortable	3.9 (2.5)	3.8 (2.3)	0.93
Found monitor straightforward to use	7.5 (3.2)	8.4 (2.8)	0.06
Found monitor cumbersome to wear...			
At home	3.8 (3.0)	3.9 (2.8)	0.97
At work	3.1 (2.8)	3.8 (2.9)	0.06
Driving	3.6 (2.9)	3.8 (2.9)	0.35
Other times	3.8 (2.9)	3.5 (2.7)	0.47
Noise of the pump disturbed [me]...			
At home	2.3 (2.5)	2.2 (2.6)	0.82
At work	2.2 (2.7)	2.6 (2.8)	0.14
Driving	1.3 (1.7)	1.6 (2.4)	0.34
Other times	1.9 (2.2)	2.3 (2.6)	0.23
Noise of the pump disturbed others	2.3 (2.5)	2.3 (2.6)	0.81
Found monitor embarrassing to wear	1.7 (2.8)	2.2 (3.0)	0.04
Monitor interfered with normal sleeping pattern	4.2 (3.3)	4.3 (3.5)	0.84

*P-value by paired t-test with two-sided alpha

### Adverse effects and outcomes of monitor wear

After wearing the monitor the first time, 19.6% reported that it stopped them from falling asleep, and 70.2% reported that the monitor awakened them from sleep (Table 3), with 8.8% reporting that the monitor disturbed them enough to make them remove it during the night. Fewer reported that the monitor disturbed them enough to make them remove it at some point during the day (5.1% and 8.5%). Nearly one out of three wearers reported pain from wearing the monitor, and 39.0% experienced skin irritation after first-wear with a slight increase (45.8%) at second wear. After first wearing the monitor, 6.8% reported experiencing bruising. After wearing the monitor the second time, though, 20.3% reported bruising.

**Table 3:** Outcomes and adverse effects of monitor wear

	**Percent after 1**^**st **^**session**	**Percent after 2**^**nd **^**session**	p-value*
Monitor stopped me from falling asleep	19.6	16.1	0.48
Monitor woke me up after falling asleep	70.2	64.9	0.41
Monitor disturbed me sufficiently to make me remove it during the day	5.1	8.5	0.32
Monitor disturbed me sufficiently to make me remove it during the night	8.8	8.8	1.0
Experienced pain from wearing the monitor	33.9	35.6	0.76
Experienced skin irritation from wearing the monitor	39.0	45.8	0.35
Experienced bruising from wearing the monitor	6.8	20.3	0.02

*P-value by McNemar's chi square test

### Poor tolerability and data acquisition

People categorized as having poor tolerability to wearing the monitor based on their responses to the Likert-scaled questions were largely the same at both monitoring sessions, with 82% agreement (kappa = 0.6, p = 0.0001). Among people who reported fair/poor health, 75% tolerated the monitor poorly, but among the participants reporting good to excellent health, 22.2% tolerated the monitor poorly (p = 0.01) (Table 4). Though not statistically significant, those with higher 24-hour BP also tended to tolerate the monitor more poorly. A higher proportion of participants categorized as having poor tolerability removed the monitor during the day during the first wear (7.7% vs 2.8%) and the second wear (14.3% vs 5.0%) (Table 5). However, neither of these differences were statistically significant (p = 0.44 and p = 0.25, respectively). A higher proportion of participants categorized as having poor tolerability removed the monitor during the night (15.4% vs 2.9%, p = 0.11) during the first wear, and a significantly higher proportion of participants categorized as having poor tolerability removed the monitor during the night (28.6% vs 2.5%, p = 0.004) during the second wear.

**Table 4:** Associations with poor tolerability to wearing monitor

Characteristic	Percent	p-value*
Age group (years)		0.27
29-44	38.9	
45-64	20.1	
>65	0	
Sex		0.81
Male	25.0	
Female	28.0	
Race		0.73
Black	30.4	
White	24.0	
Other	0	
Education level		0.53
Some high school	0	
High school graduate	0	
Some college	26.3	
College graduate	32.0	
Self-reported health		
Excellent, very good, good	22.2	0.01
Fair/poor	75.0	
Smoker		0.98
Yes	25.0	
No	24.4	
BMI		0.36
Normal (<25 kg/m^2^)	36.4	
Overweight (25-29 kg/m^2^)	13.3	
Obese ( ≥30 kg/m^2^)	30.4	
Arm circumference		0.47
<35 cm	23.5	
≥35 cm	33.3	
Married or living with partner		
Yes	21.9	0.31
No	35.3	
24-hour systolic BP ≥ 130 mm Hg		0.56
Yes	27.9	
No	16.7	
24-hour diastolic BP ≥ 80 mm Hg		0.37
Yes	29.7	
No	16.7	

BMI, body mass index

*P-value by chi-square

**Table 5:** Associations of poor tolerability with removal of monitor and inadequate data acquisition

	Poor tolerability to wearing monitor		
	
	Yes (%)	No (%)	p-value
Day removal, first wearing	7.7	2.8	0.44
Night removal, first wearing	15.4	2.9	0.11
Day removal, second wearing	14.3	5.0	0.25
Night removal, second wearing	28.6	2.5	0.004
Inadequate data for daytime, first wearing	15.4	2.8	0.10
Inadequate data for nighttime, first wearing	23.1	19.4	0.78
Inadequate data for entire 24 hours, first wearing	23.1	19.4	0.78
Inadequate data for daytime, second wearing	21.4	2.5	0.02
Inadequate data for nighttime, second wearing	42.9	22.5	0.14
Inadequate data for entire 24 hours, second wearing	50.0	22.5	0.05

At the first monitoring session the number of ambulatory BP measurements during daytime wear was inadequate among 15.4% of participants categorized as having poor tolerability and 2.8% (p = 0.10) among those not categorized as such (Table 5). The percent of participants with inadequate measurements over the entire 24-hours during the first monitoring session was similar between the two groups. At the second monitoring, however, the percents of participants with inadequate measurements during daytime wear were 21.4% vs 2.5% (p = 0.02) and over 24-hours were 50.0% vs 22.5% (p = 0.05).

## Discussion

Overall, participants in this study seemed to have little difficulty with using the Oscar 2 ambulatory BP monitor. As has been found in other studies of tolerability of ambulatory BP monitoring, the greatest disturbance from wearing the monitor was interference with sleep [9-11]. Two-thirds of wearers reported that the monitor awakened them from sleep. Nonetheless, most participants continued to wear the monitor at night. We found no studies with which to make a direct comparison about the proportion of participants who reported actually removing the monitor. Our questions about monitor removal were modified from a prior study's questionnaire [8], and we found no other study containing similar items. In that study of pregnant women undergoing ABPM using a SpaceLabs 90207 (SpaceLabs Medical, Inc, Redmond, WA) monitor, 15% discontinued their monitoring session [8]. However, the results may not be comparable to ours due to the different population and the slightly different questions used.

We found that the most commonly reported adverse physical effects were pain and skin irritation. Bruising was also reported, although less frequently. It is possible that the second monitoring session resulted in more bruising because it occurred so shortly after the first session. The proportion of participants with inadequate ambulatory BP data was also higher data at the second monitoring. While there may be a need for ambulatory BP monitoring on consecutive weeks in research studies, it is less likely to be used in this manner for clinical care. Other studies have found that approximately 90 to 95% of patients undergoing ambulatory BP monitoring for clinical reasons would be willing to have a repeat monitoring session if so advised [10,12].

Not surprisingly, we found that people with poor tolerability to wearing the monitor were more likely to remove it, particularly during sleep. However, at the first monitoring session, 24-hour data acquisition was adequate in nearly three-fourths of the participants regardless of whether classified as having poor tolerability or not. This information can be useful for investigators planning research studies. It may be difficult to know who will tolerate the monitor poorly, but we did find that people whose self-reported health status was fair or poor were more likely to be categorized as having poor tolerability. However, this may simply reflect a "rating bias" whereby people who rate their health negatively may rate everything negatively. Our study also may overestimate true tolerability since our sample consisted of voluntary participants in a research study who received monetary incentive for wearing the monitor on two occasions.

To our knowledge, this is the first study to examine the tolerability of ambulatory BP monitoring using the Oscar 2 monitor with its specially designed Orbit cuff. Adverse effects of wearing this monitor seem similar to those described with other monitors. In a study of acceptability of wearing the Mobil-O-Graph (IEM GmbH, Stolberg, Germany) oscillometric ambulatory BP monitor, 55% of patients reported interference with sleep, 41% reported pain, and 17% had a local skin reaction [12]. In an examination of acceptance of ambulatory monitoring using a SpaceLabs 90207 device, 20% of patients reported that the session was uncomfortable [13]. Other studies of the SpaceLab 90202 device found that 30% to 36% reported disturbed sleep, up to 20% reported pain, and 19% to 34% reported that monitoring disturbed their activities [9,14].

Another strength of this study is its assessment of tolerability on two occasions, providing some estimate of the reproducibility of a questionnaire designed to measure people's experience with wearing an ambulatory BP monitor. We are aware of no other study that has done this type of evaluation. Perhaps most importantly, we analyzed the effects of tolerability on the number of measurements one can expect to obtain from studies of participants in which ambulatory BP monitoring is used. We believe this is useful information for researchers as well as clinicians who use ambulatory BP monitoring.

Our study is limited by a small sample size. It is likely that some of the factors we examined (e.g., elevated BP, greater arm circumference) would be statistically significant with a larger sample. Additionally, because this study was of research participants who volunteered (and were paid) to participate, the findings may not be generalizable outside the research setting. Tolerability was also based on people's self reports, and reported side effects (e.g., bruising) were not verified objectively.

## Conclusions

Despite the above limitations, it is clear that ambulatory BP monitoring using current devices, including the Oscar 2 monitor, is not a procedure without some inconveniences and some minor transient adverse effects. It thus does not come as a surprise that ambulatory monitoring has been found to be the least patient-preferred method of measuring BP (after home monitoring and office measurement) [15]. However, studies show that patients also can appreciate the value of the information provided by ambulatory BP monitoring [13]. The data on potential adverse effects as well as the likelihood of disturbances of activities (especially sleep) should be explained to research subjects (or patients) along with the importance of the blood pressure information to be obtained from ambulatory BP monitoring so that people undergoing monitoring understand both the risks and benefits.

## Competing interests

On March 24, 2011, AJV began serving on the Medical Advisory Board for SunTech Medical.

## Authors' contributions

AJV conceived of the study, conducted the analyses, drafted and revised the manuscript. KL fitted participants with the 24-hour ambulatory blood pressure monitors, administered questionnaires, collected data, entered data, and provided critical review of the manuscript. ALH assisted in conceiving the study and interpreting the data and provided critical review and revisions to the manuscript. All authors read and approved the final manuscript.

## Appendix - Questionnaire

POST-AMBULATORY BP MONITORING QUESTIONNAIRE

*For the following questions, please circle the answer that corresponds to your response on a scale from 0 to 10:*

0 = "Not at all"   5 = "Somewhat"   10 = "Extremely"

1. Did you find the monitor heavy?

0   1   2   3   4   5   6   7   8   9   10

2. Did you find the monitor comfortable to wear?

0   1   2   3   4   5   6   7   8   9   10

3. Did you find the monitor straightforward to use?

0   1   2   3   4   5   6   7   8   9   10

4. Did you find the monitor cumbersome to wear...

At home?

0   1   2   3   4   5   6   7   8   9   10

At work?

0   1   2   3   4   5   6   7   8   9   10

Driving?

0   1   2   3   4   5   6   7   8   9   10

At other times?

0   1   2   3   4   5   6   7   8   9   10

5. Did the noise of the pump disturb you...

At home?

0   1   2   3   4   5   6   7   8   9   10

At work?

0   1   2   3   4   5   6   7   8   9   10

Driving?

0   1   2   3   4   5   6   7   8   9   10

At other times?

0   1   2   3   4   5   6   7   8   9   10

6. Did the noise of the pump disturb others?

0   1   2   3   4   5   6   7   8   9   10

7. Did you find the monitor embarrassing to wear?

0   1   2   3   4   5   6   7   8   9   10

8. Did you find the monitor interfered with your normal sleeping pattern?

0   1   2   3   4   5   6   7   8   9   10

*Please circle your response to the next questions*.

9. If your sleep was disturbed, did the monitor stop you from falling asleep?

Yes   No   Sleep was not disturbed

10. If your sleep was disturbed, did the monitor wake you up after you had fallen asleep?

Yes   No   Sleep was not disturbed

11. Did the monitor disturb you sufficiently to make you remove it during the day?

Yes   No

12. Did the monitor disturb you sufficiently to make you remove it during the night?

Yes   No

13. Did you experience pain from wearing the monitor?

Yes   No

14. Did you experience skin irritation from wearing the monitor?

Yes   No

15. Did you experience bruising from wearing the monitor?

Yes   No

THANK YOU FOR COMPLETING THIS QUESTIONNAIRE!

## Pre-publication history

The pre-publication history for this paper can be accessed here:

http://www.biomedcentral.com/1471-2288/11/59/prepub
